# Evaluating A Brief Telehealth Positive Parenting Intervention: A Randomized Controlled Trial 

**DOI:** 10.1007/s11121-026-01894-3

**Published:** 2026-03-21

**Authors:** Brianna T. Ricker, John L. Cooley, Victoria E. Dennis, Brooke E. Streicher, Tarrah B. Mitchell, Caroline Cummings, Jonathan Singer

**Affiliations:** 1 Department of Psychological Sciences, Texas Tech University, Box 42051, Lubbock, TX 79409-2051 USA; 2 School of Special Education, School Psychology, and Early Childhood Studies, University of Florida, Gainesville, FL USA; 3 Prevention and Intervention Network, College of Education, University of Florida, Gainesville, FL USA; 4 Department of Psychiatry, University of Florida, Gainesville, FL USA; 5 Department of Pharmacology and Neuroscience, Texas Tech University Health Sciences Center, Lubbock, TX USA; 6 Garrison Institute on Aging, Texas Tech University Health Sciences Center, Lubbock, TX USA

**Keywords:** Parenting intervention, Telehealth, Positive parenting, Universal intervention

## Abstract

Additional work is needed to increase the accessibility and scalability of parenting interventions. Selected Positive Parenting Program (i.e., Triple P) is a brief, evidence-based parenting intervention, but the efficacy of telehealth delivery has not yet been tested. This three-arm randomized controlled trial evaluated the feasibility, acceptability, and preliminary efficacy of Selected Triple P delivered as a universal intervention through telehealth. We also investigated whether an active discussion component administered by mental health providers improved outcomes. This clinical trial was retrospectively registered on 3/3/2025 (NCT06865183). Participants included 97 parents/caregivers of children ages 2–12 randomly assigned to one of three conditions: intervention-as-usual (90-min condition; *n* = 32), intervention without an active discussion (60-min condition; *n* = 24), or waitlist control (*n* = 41). Parents completed assessments at baseline and 2-months post-intervention. No baseline differences were found between conditions on any demographic or outcome variables. Parents in both intervention conditions reported high levels of satisfaction and acceptability, with no significant differences observed between groups. Results indicated that, compared to the control group, parents in the 90-min condition reported significant increases in positive reinforcement and positive parenting, along with reductions in parental hostility; decreases in negative parenting also approached significance. Although changes were not statistically significant for parents in the 60-min condition relative to the control group, there were no significant differences between intervention conditions across these outcomes. Current findings have important implications for the scalability and dissemination of universal parenting interventions, as well as strategies for increasing access to mental health services among families. ClinicalTrials.gov 3/3/2025; Registration Number: NCT06865183. Trial Registration.

**Supplementary Information:**

The online version contains supplementary material available at 10.1007/s11121-026-01894-3.

Parents^1^ play a central role in child development. Accordingly, positive parenting—which includes warmth, encouraging good behavior (e.g., following directions, being kind to others), planning ahead, managing misbehavior, and teaching new skills (Sanders, 2008)—is associated with adaptive youth outcomes (e.g., Morris et al., 2017) and can buffer against stressors, such as socioeconomic disadvantage (Brown et al., 2020). As such, parenting interventions may be a key strategy for promoting child well-being, with evidence supporting their effectiveness in promoting positive parenting and reducing children’s emotional and behavioral difficulties (Morris et al., 2020; Salari & Enebrink, 2018).

Despite the effectiveness of many parenting interventions, there is a well-documented gap between the number of families needing mental health services and those who can access them (Kazdin, 2019; Kohn et al., 2018). Notably, recent trends suggest that youth mental health is worsening (Racine et al., 2021), highlighting an increasing need for scalable interventions. However, the dominant model of mental health service delivery may be contributing to the current treatment gap (Kazdin, 2019). That is, most interventions involve highly trained (i.e., a master’s- or doctoral-level) mental health professionals providing individual treatment in outpatient settings (Kazdin, 2019). Although effective, this structure, along with a shortage of providers, has resulted in long waitlists for services (Butryn et al., 2017; Kazdin, 2019). Further, this model creates several logistical barriers, including access to transportation and childcare, which contribute to low levels of parental engagement in many existing interventions (Baker et al., 2011). Therefore, there is an urgent need to a need to evaluate alternative methods of program delivery and dissemination to increase accessibility and engagement (Kazdin, 2019).

One promising approach to address these barriers is the implementation of universally available, brief parenting interventions via telehealth (Salari & Enebrink, 2018; Schleider et al., 2020). Currently, most parenting interventions are delivered at the indicated (i.e., offered to individuals with clinically significant problems) or selective (i.e., offered to individuals who are experiencing a known risk factor) levels (Salari & Enebrink, 2018), meaning that services are not accessible until problems have emerged. Universal interventions (i.e., offered to all individuals), by contrast, may improve child outcomes by enhancing positive parenting more broadly, which might prevent the development of more severe emotional and behavioral issues that require intensive intervention (Salari & Enebrink, 2018).

Further, telehealth offers a scalable and accessible solution for interventions by removing logistical barriers (Ros-DeMarize et al., 2021) and has proven to be effective in improving both parenting (Flujas-Contreras et al., 2019) and child outcomes (Florean et al., 2020). Importantly, telehealth also has implications for program dissemination, in that parents who live in rural areas or who lack access to local mental health providers can still receive services as long as they have internet access.

Moreover, many parenting interventions, including those that are offered universally, span several months (Morris et al., 2020), which can exacerbate logistical difficulties (Schleider et al., 2020). Research suggests that adding additional components to parenting interventions does not necessarily improve their effectiveness (e.g., Bakermans-Kranenburg et al., 2003). For example, in a meta-analysis of early parenting interventions, shorter interventions (i.e., fewer than 5 sessions) were just as effective as those lasting between 5 and 16 sessions—and even more effective than those over 16 sessions—in improving parental sensitivity and attachment (Bakermans-Kranenburg et al., 2003). Similarly, a meta-analysis examining components of parenting interventions found that core strategies such as positive reinforcement, praise, and natural/logical consequences were linked to stronger improvements in child behavior, including in prevention contexts (Leijten et al., 2019). Together, this literature indicates that brief, targeted parenting programs can achieve meaningful improvements in parenting and child behavior while reducing logistical barriers to participation. Offering these interventions universally via telehealth may further enhance accessibility by bypassing structural barriers in traditional service delivery, ultimately increasing the reach and impact of evidence-based parenting support.

## Selected Triple P

The Positive Parenting Program (Triple P) is a multi-level intervention program that targets parenting skills, knowledge, and self-efficacy to prevent emotional and behavioral problems in children (Sanders, 2012, 2023). It is based on principles of social and operant learning theories, such that parents are taught how to increase the occurrence of positive child behaviors while reducing negative behaviors (Sanders, 2012, 2023). An emphasis is also placed on increasing the use of positive parenting behaviors (e.g., praise) with hopes of reducing less adaptive parenting behaviors (e.g., corporal punishment; Sanders, 2012, 2023).

The focus of the current study is Selected Triple P, which is a brief seminar series designed for parents of children ages 12 and under (Sanders et al., 2009). The intervention consists of three 60-min seminars followed by 30-min question-and-answer periods. Seminars can be delivered in groups of up to 200 parents (Sanders et al., 2009), allowing for broad dissemination. The brief format and opportunity to adapt the intervention for telehealth delivery further enhances its accessibility. Notably, the 30-min discussion component allows parents to ask questions and receive feedback from mental health providers (Sanders et al., 2009). Some researchers have emphasized the importance of parents’ ability to discuss and practice skills with a provider for intervention efficacy (Morris et al., 2020). However, other parenting interventions have been offered online—without this component—and have still demonstrated positive effects on child outcomes and parental mental health (for a review, see Thongseiratch et al., 2020). Additionally, interventions that do not require a mental health professional for administration have the potential for greater scalability (e.g., Love et al., 2013).

Several randomized controlled trials (RCTs) have demonstrated that Selected Triple P, when attended in person, significantly improves parenting behaviors. In a pilot study, 244 parents of children ages 4–7 years in Australia were randomly assigned to attend only the first seminar, attend all three seminars, or a control group (Sanders et al., 2009). Parents in both intervention conditions reported fewer dysfunctional parenting behaviors (e.g., laxness, over-reactivity, and verbosity). Another RCT conducted in Indonesia with 143 parents of children ages 2–12 found that participation in the entire seminar series resulted in greater parental confidence and a reduction in dysfunctional parenting behaviors post-intervention, when compared to parents in the control group (Sumargi et al., 2015). A more recent RCT with 124 parents of children ages 2–12 in Greece found that those who participated in the seminar series reported engaging in fewer dysfunctional parenting behaviors post-intervention; however, there was no significant between-group difference in parental confidence over time (Foskolos et al., 2023).

Open trials provide additional evidence that Selected Triple P is effective in improving parenting outcomes. One pilot open trial administered one seminar to 30 Indonesian parents with children ages 2–12 and found reductions in dysfunctional parenting behaviors 3 weeks post-intervention (Sumargi et al., 2014). A subsequent open trial of 27 parents with children ages 2–6 years in Canada found that participation in Selected Triple P resulted in using less corporal punishment and more non-physical punishment post-intervention; however, there was no change in parents’ use of non-punitive responses (Gonzalez et al., 2019). One Selected Triple P seminar was delivered to parents who were experiencing homelessness in another investigation, and parents reported that they found the content to be interesting, helpful, and relevant (Haskett et al., 2018). Finally, a meta-analysis revealed that Selected Triple P was associated with significant small to moderate effects on parental satisfaction and efficacy (*d* = 0.24) and effective parenting behaviors (*d* = 0.47) post-intervention (Sanders et al., 2014).

### The Current Study

Selected Triple P addresses several limitations of previous parenting interventions in that it is brief (i.e., 3 sessions) and is designed to be delivered to large groups. Although not yet tested empirically, the format of the seminars makes them conducive to administration via telehealth, which would reduce logistical barriers for families. Therefore, the purpose of the three-arm RCT was to investigate the feasibility, acceptability, and preliminary efficacy of Selected Triple P as a universal intervention delivered through telehealth. Parents were randomized to a standard seminar format including an active discussion component facilitated by a mental health provider, a seminar format without active discussion, or a waitlist control condition. Virtual delivery of the intervention may reduce common logistical barriers and increase accessibility for families. As such, feasibility and acceptability were assessed through parents’ engagement with the intervention when delivered via telehealth (i.e., seminar attendance) and self-reported satisfaction and acceptability. Changes in parenting behavior, parental knowledge, parental hope, readiness for change, and parental self-efficacy were examined as indicators of preliminary efficacy. A secondary aim was to investigate whether the inclusion of an active discussion component facilitated by a mental health provider impacted parents’ satisfaction and acceptability and their post-intervention changes in these outcomes. The discussion component was designed to allow parents to ask questions, receive individualized feedback, and clarify skill implementation (Sanders et al., 2009), which may enhance engagement, understanding, and application of strategies beyond didactic seminar delivery alone. As such, it is important to examine whether these interactive elements confer added benefit, or whether access to the seminar content alone is sufficient to promote change.

Based on previous research (Haskett et al., 2018; Ros-DeMarize et al., 2021), we hypothesized that the intervention would be feasible and acceptable when delivered via telehealth, as indicated by parents’ seminar attendance and self-reported satisfaction with the virtual format and seminar content. We also anticipated that parents in the intervention conditions would demonstrate improvements in positive parenting (e.g., warmth, support), reductions in negative parenting (e.g., hostility), and increases in parental knowledge (Winter et al., 2012), parental hope (Cole & Molloy, 2023), parental self-efficacy (Wittkowski et al., 2016), and readiness for change (Sung et al., 2023) compared to those in the control group. To our knowledge, no studies have investigated whether an active discussion component is necessary for intervention efficacy, so no a-priori hypothesis was made regarding its impact.

## Method

### Participants

To be eligible, parents needed at least one child ages 2–12 (e.g., Foskolos et al., 2023) who was not in mental health treatment or had not started psychiatric medication within the past month. Any caregiver (e.g., parent, step-parent, grandparent) was eligible if the child lived with them at least 1 night every 2 weeks. Parents were not eligible if they were currently participating in therapy or counseling for themselves. Parents needed to be English-speaking, have access to the internet, live in the State of Texas, and complete the baseline survey. Intervention parents had to attend at least one seminar to be included in intervention efficacy analyses^2^.

**Fig. 1:**
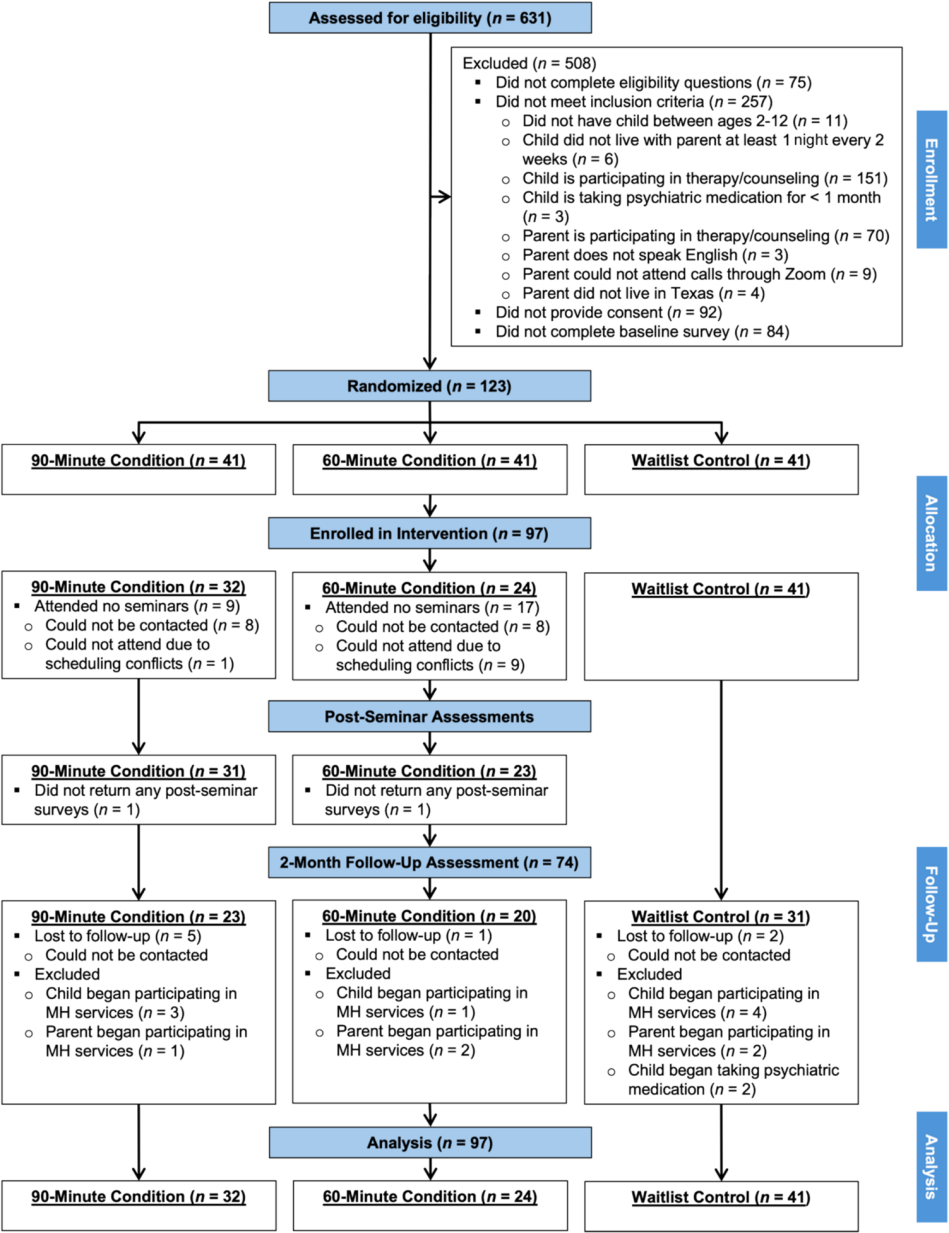
CONSORT Flow Diagram. MH = Mental health

Of the 631 individuals assessed for eligibility, 508 were excluded (see Fig. 1 for the CONSORT flow diagram). A total of 123 parents met inclusion criteria, provided consent, and were randomized to the 90-min condition (i.e., intervention-as-usual; *n* = 41), the 60-min condition (i.e., intervention without active discussion; *n* = 41), or the waitlist control group (*n* = 41) using block randomization. After random assignment, three parents from the 60-min condition withdrew due to scheduling conflicts or medical issues. Twenty three parents assigned to an intervention condition (90-min condition: *n* = 9; 60-min condition: *n* = 14) did not attend any seminars and were excluded from efficacy analyses, resulting in a final sample size of 97 parents (90-min: *n* = 32; 60-min: *n* = 24; waitlist control: *n* = 41). A chi-square test confirmed that the distribution of participations across conditions did not differ from chance, χ^2^, (2, 97) = 4.47, *p* = .11.

Participant demographic information is provided in Tables S1–S3. Overall, 72.2% of participants were biological mothers (*n* = 70), 19.6% were biological fathers (*n* = 19), 3.1% were adoptive mothers (*n* = 3), 2.1% were stepmothers (*n* = 2), and 3.1% were other caregivers (e.g., grandparent, aunt; *n* = 3). Most parents (71.1%) were White and non-Hispanic/Latine (73.2%). According to parents, 62.9% lived outside of the city where the research team was located and 13.4% resided in rural counties (Health Resources & Services Administration, n.d.). On average, parents reported 1.90 (*SD* = 0.47) adults and 1.99 (*SD* = 1.06) children living in the household. The average household income was $100,310 (*SD* = $81,328, Range = $3,600—$500,000). Children (60.8% boys) ranged in age from 2–12 years (*M* = 6.60 years, *SD* = 3.21). Importantly, there were no differences between conditions on any demographic variables.

### Procedures

Study flyers were distributed through local preschools, elementary schools, community organizations, and a university’s announcement system in the same city as the research team, as well as through social media groups (i.e., Facebook) that included parents from across Texas. Interested parents accessed an eligibility survey through a QR code. After providing consent, eligible parents were asked to complete a baseline survey online to enroll. Parents responded to survey questions about one child (ages 2–12) with whom they experienced the most difficulties and answered questions about the same child on all subsequent surveys.

After completing the baseline survey, parents were randomly assigned to conditions using block randomization. Block sizes (range: 3–9) were predetermined via a random number generator, and the allocation sequence within each block was randomly selected (Efird, 2011). Recruitment was ongoing and seminars were scheduled when there were at least 10 parents in each condition to maintain a group setting during the seminars and to reduce the amount of time between enrollment and when seminars were offered. A round of three seminars was offered for each of the three enrollment cohorts, along with a make-up round of seminars—separately for the 90- and 60-min conditions—for parents who missed any of the seminars. Parents who were initially unable to attend were contacted and informed each time the seminars were offered again, so there were multiple opportunities to participate in the intervention.

At the end of each seminar, parents in both intervention conditions were asked to complete a satisfaction survey and were compensated $5 for each survey they completed (up to $15 total). All parents were contacted approximately 2 months after attending the final seminar to complete a follow-up survey, for which they were compensated $25. Parents in the waitlist control condition were contacted approximately 2 months after the last seminar was offered to those assigned to the intervention conditions in their enrollment cohort.

### Intervention Procedures

The first seminar, *The Power of Positive Parenting*, introduces five core principles of positive parenting and teaches strategies such as praise, effective commands, logical consequences, planned ignoring, and time-out techniques (Sanders & Turner, 2012). The second seminar, *Raising Confident, Competent Children*, focuses on fostering children’s social competence by building communication, self-esteem, problem-solving, and independence skills. The final seminar, *Raising Resilient Children*, helps parents support children’s emotion regulation by teaching them to identify, express, and manage emotions, develop a positive outlook, and build coping skills (Sanders & Turner, 2012). Although the specific focus of each seminar varied, all sessions consistently emphasized core parenting strategies such as praise and positive reinforcement, proactive parenting, and maintaining a warm parent–child relationship.

Attendance was tracked at the beginning of the seminar and then every 10 min by a member of the research team. The seminars were co-led by two master’s-level graduate students in a Clinical Psychology Ph.D. program who are fully accredited in the intervention and were supervised by a licensed clinical psychologist who was also fully accredited. Every seminar across both conditions was video recorded^3^.

#### 90-Minute Condition

Seminars were conducted live by two co-leaders. They presented seminar content for approximately 60-min using PowerPoints developed by Triple P, followed by a 30-min question-and-answer period. Parents were able to ask questions throughout each seminar by unmuting their microphone or using the chat feature. Co-leaders also encouraged participation by asking parents to respond to polls and discussion questions.

#### 60-Minute Condition

Seminars were pre-recorded to ensure standardization of the material and to create an environment focused on receiving information rather than active participation. During the seminars , participants could not unmute their microphones and could only message the group leader using the chat feature. After an introduction, the leader played the pre-recorded seminar and remained in the meeting to record attendance.

### Measures

#### Preliminary Outcomes

### Satisfaction

Parents in intervention conditions completed satisfaction measures after each seminar and at the 2-month follow-up.

### Post-Seminar Satisfaction

Parents completed 8 items (e.g., “Overall, how would you rate the content of the seminar?”) from the Parent Satisfaction Survey (PSS; Sanders & Turner, 2012) after each seminar. Two items were removed for relevance, and an additional item was added to assess satisfaction with the telehealth format (e.g., “How satisfied are you with the opportunity to attend the seminar virtually?”). The items were averaged after each seminar, and then across all three seminars to create an overall measure of satisfaction. Internal consistency was good to excellent after each seminar in the current sample (αs = .86—.93). The telehealth satisfaction item was analyzed separately.

### Global Satisfaction

At the 2-month follow-up, parents completed the 8-item (e.g., “To what extent did our program meet your needs?”) Client Satisfaction Questionnaire (Larsen et al., 1979). Items were averaged. Internal consistency was good in the current sample (α = .89).

#### Acceptability

Parents completed a modified version of the Treatment Acceptability Rating Form-Revised (TARF-R; Reimers et al., 1992) after each seminar. A subset of 9 items^4^ (e.g., “How likely are these strategies going to be effective for your child?”) were selected to minimize redundancy of items from other measures and because some of the items from the original measure were not relevant for the current study. Items were averaged after each seminar, and then across all three seminars to create an overall measure of acceptability. Internal consistency was adequate (αs = .75—.80).

### Primary Intervention Outcomes

#### Parenting Behaviors

Parents completed the 34-item Multidimensional Assessment of Parenting Scale (MAPS; Parent & Forehand, 2017) at baseline and the 2-month follow-up. The MAPS includes proactive parenting (e.g., “I avoid struggles with my child by giving clear choices”), positive reinforcement (e.g., “If my child cleans his room, I will tell him/her how proud I am”), warmth (e.g., “My child and I hug and/or kiss each other”), and supportiveness (e.g., “I encourage my child to talk about his/her troubles”) subscales, which comprise broadband positive parenting. Other subscales include hostility (e.g., “I argue with my child”), physical control (e.g., “I spank my child with my hand when he/she has done something wrong”), and lax control (e.g., “I feel that getting my child to obey is more trouble than it’s worth”), which comprise broadband negative parenting. The individual subscales and broadband measures were used. Items were summed. Internal consistency was good for positive (αs = .81—87) and negative parenting (αs = .86—87) at baseline and follow-up.

Parents also completed the 8-item (e.g., “Telling your child he or she is not as good as other children”) Parent Psychological Control Measure (Hart et al., 1998) that is based off the Psychological Control Scale – Youth Self Report (Barber, 1996) at baseline and the 2-month follow-up. Scores were summed. Internal consistency was good baseline and the 2-month follow-up in the current sample (αs = .80—.86).

#### Parental Knowledge

Parents completed the 16-item (e.g., “If you want a child to say “please” and “thank you”, it is probably most important to…”) Brief Version of the Knowledge of Parenting Strategies Scale (Kirkman et al., 2018) at baseline and the 2-month follow-up. Parents’ scores reflected the percentage of correct answers. Due to the dichotomous nature of the items, internal consistency was not calculated (Cohen et al., 2003).

#### Parental Hope

Parents completed the 5-item (e.g., “I can think of specific ways to be a good parent”) Hope for Parenting Scale (Cole et al., 2021) at baseline and the 2-month follow-up. Scores were summed. Internal consistency was adequate at baseline and the 2-month follow-up in the current sample (αs = .76—78).

#### Readiness for Change

Parents completed the Readiness for Change Ruler (Sung et al., 2023) at baseline and the 2-month follow-up. The measure consists of 3 items, which were slightly adapted to assess for readiness to improve parenting skills (e.g., “At this moment, how important is working toward improving your parenting skills?”). Items were summed. Internal consistency was good at baseline and follow-up in the current sample (αs = .81- .85).

#### Parental Self-Efficacy

Parents completed the 5-item (e.g., “I am able to do the things that will improve my child’s behavior”) Brief Parental Self-Efficacy Scale (Woolgar et al., 2023) at baseline and the 2-month follow-up. Scores were summed. Internal consistency was good at baseline and the 2-month follow-up in the current sample (αs = .81—.82).

### Data Analysis

Preliminary analyses and descriptive statistics were estimated within SPSS (Version 29). A series of ANOVAs and chi-square (χ^2^) tests were estimated to assess for baseline differences between conditions on outcome and demographic variables. Independent sample *t*-tests were estimated to determine whether there were differences in seminar attendance and measures of satisfaction and acceptability between the two intervention conditions.

A series of multilevel models were then estimated within SAS (On Demand for Academics) using PROC MIXED to evaluate intervention effects, such assessments at Level 1 were nested within persons at Level 2. A time lag variable was created to reflect individual participants’ duration in months between the baseline and 2-month follow-up assessments; on average, participants had a lag of 3.21 months between assessments (*SD* = 0.68, Range = 2.17—5.60). Model parameters were estimated using restricted estimation maximum likelihood (REML) to account for missing outcome data at Times 1 (1%) and 2 (27.8%).

Dummy-coded variables (waitlist = 0; intervention = 1) were created to allow for comparisons between conditions. In the first model, two dummy-coded variables were created to allow for comparisons between those in an intervention condition and those in the control group. A fixed linear effect of time, intervention group variables, and time x intervention group interactions were added to the model. Wald tests were used to assess the significance of model parameters. Estimates were also converted to partial Cohen’s *d* values (small = 0.2, medium = 0.5, large = 0.8). Next, the same series of models were estimated with two alternative dummy-coded variables to allow for comparison between the two intervention conditions. Outcome analyses were restricted to parents assigned to an intervention condition and attended at least one seminar, whereas descriptive analyses of attendance included all randomized participants.

### Power Analysis

G*Power was utilized to determine the necessary sample size to have adequate power (i.e., 80%; Erdfelder et al., 1996). An a priori power analysis was conducted with *f* = 0.18 based on the minimum effect size observed in a recent study utilizing the Triple P seminar series (Foskolos et al., 2023). Several models were estimated using three groups and two measurement points with varying strengths of the correlations between measures over time (*r*s = .30 – .70). To have adequate power (i.e., 1-β = 0.8) to detect significant within-between interactions (*p* < .05), a total sample size between 48 and 108 (i.e., between 16 and 36 per condition) was required. A post-hoc power analysis was also conducted to calculate the observed power for the final sample size of 97 participants. Effect sizes in the current study ranged from *d* = 0.13 to *d* = 0.51. Corresponding power ranged from 1–β = 0.14 to 0.99, indicating that the study was sufficiently powered to detect effects where *d* > 0.32—that is, moderate to large effects—but underpowered to detect small effects.

## Results

Preliminary analyses showed no significant differences between conditions on any demographic variables (see Table S1–S3). As seen in Table S4, a series of one-way ANOVAs revealed no significant baseline differences in any outcome variables between conditions.^5^

### Feasibility and Acceptability

Among all parents randomized to an intervention condition (including those who did not attend any seminars), parents in the 90-min condition attended 1.95 (*SD* = 1.26) seminars on average; 53.7% (*n* = 22) attended all three seminars, 9.8% (*n* = 4) attended two seminars, and 14.6% (*n* = 6) attended one seminar. Parents in the 60-min condition attended 1.66 (*SD* = 1.42) seminars on average; 50% (*n* = 19) attended all three seminars, 2.6% (*n* = 1) attended two seminars, and 10.5% (*n* = 4) attended one seminar. Notably, there was no difference in seminar attendance between conditions, *t*(77) = 0.97, *p* = .33.

Overall, parents reported high levels of satisfaction with the seminars (see Table S5). There were no significant differences in satisfaction ratings between the two conditions *t*(50) = 0.67, *p* = .25, *d* = 0.19. Parents also reported high levels of satisfaction with the telehealth format, with no significant difference between conditions, *t*(36) = 1.04, *p* = .15, *d* = 0.34 (see Table S5). On a measure of global satisfaction, parents again reported high levels of satisfaction, with no differences between conditions, *t*(39) = 0.81, *p* = .21, *d* = 0.25 (see Table S5). Acceptability scores were slightly lower compared to satisfaction ratings, but were still high overall. As seen in Table S5, there was not a significant difference in acceptability ratings between conditions, *t*(50) = 0.89, *p* = .19, *d* = 0.25.

### Intervention Efficacy

**Fig. 2:**
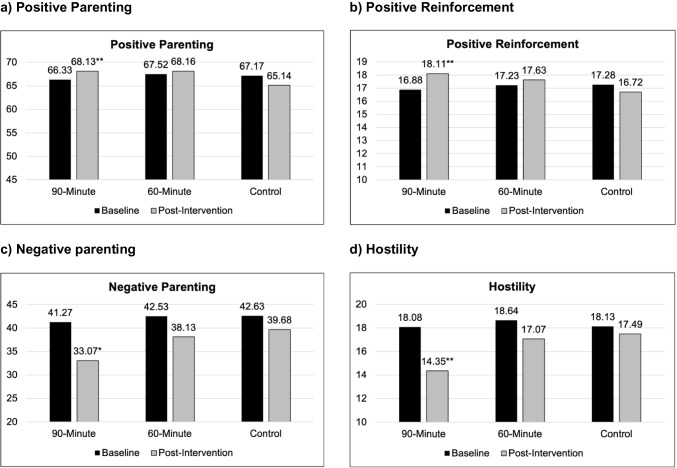
Changes in Parenting from Baseline to Post Intervention. **a**) Positive Parenting. **b**) Positive Reinforcement. **c**) Negative parenting. **d**) Hostility. Note. ** *p* < .05, * *p* = .06

As seen in Table 1, there was a significant increase in overall positive parenting from baseline to follow-up for the 90-min condition compared to the waitlist control condition (*d* = 0.51; see Fig. 2a). Change in positive parenting for the 60-min condition compared to the control condition was not significant (*d* = 0.37); however, subsequent analysis showed that there was also not a significant difference in change across the 90-min and 60-min conditions, *b* = 0.36, *SE* = 0.53, *p* = .50, *d* = 0.16. Parents in the 90-min condition also reported significantly greater increases in positive reinforcement than the control condition (*d* = 0.66; see Table 1 and Fig. 2b). Although change in positive reinforcement did not reach statistical significance for the 60-min condition relative to the control group (*d* = 0.37), there was not a significant difference between the 90- and 60-min conditions, *b* = 0.26, *SD* = 0.19, *p* = .18, *d* = 0.31. There were no significant differences in change in other dimensions of positive parenting behavior between any of the conditions *(p*s > .11; *d*s = 0.02—0.36)*.*

**Table 1:** Effects of Intervention on Positive Parenting Behaviors

	**Random Intercept**				**Fixed Linear Time Slope**			
	*b*	*SE*	*p*	*d*	*b*	*SE*	*p*	*d*
**Positive Parenting**								
Intercept	**67.17**	**0.99**	** < .001**	**11.89**	––	––	––	––
Time	––	––	––	––	–0.63	0.37	.10	–0.39
90-Min Condition	–0.85	1.49	.57	–0.10	**1.19**	**0.53**	**.03**	**0.51**
60-Min Condition	0.34	1.63	.83	0.04	0.83	0.53	.12	0.37
**Positive Reinforcement**								
Intercept	**17.28**	**0.34**	** < .001**	**8.80**	––	––	––	––
Time	––	––	––	––	–0.17	0.13	.20	–0.30
90-Min Condition	–0.40	0.51	.44	–0.14	**0.56**	**0.19**	**.004**	**0.66**
60-Min Condition	–0.05	0.56	.93	–0.02	0.30	0.19	.12	0.37
**Parental Warmth**								
Intercept	**12.91**	**0.25**	** < .001**	**8.02**	––	––	––	––
Time	––	––	––	––	–0.08	0.13	.57	–0.09
90-Min Condition	–0.07	0.38	.86	–0.03	0.21	0.19	.27	0.17
60-Min Condition	0.56	0.41	.17	0.22	0.08	0.19	.68	0.06
**Parental Support**								
Intercept	**13.00**	**0.25**	** < .001**	**9.11**	––	––	––	
Time	––	––	––	––	–0.03	0.10	.78	–0.07
90-Min Condition	–0.06	0.37	.88	–0.03	0.13	0.14	.36	0.21
60-Min Condition	–0.25	0.41	.55	–0.11	0.01	0.14	.93	0.02
**Proactive Parenting**								
Intercept	**24.01**	**0.46**	** < .001**	**8.84**	––	––	––	––
Time	––	––	––	––	–0.34	0.19	.07	–0.40
90-Min Condition	–0.29	0.69	.67	–0.07	0.32	0.27	.24	0.26
60-Min Condition	0.01	0.75	.99	0.00	0.43	0.27	.11	0.36

Waitlist control group is the reference. Bold estimates represent statistically significant paths (*p* < .05). *d* = partial Cohen’s *d*

As seen in Table 2, compared to the waitlist control condition, the 90-min condition exhibited reductions negative parenting from baseline to follow-up that approached significance (*p* = .06, *d* = –0.43; see Fig. 2c). Again, for the 60-min condition, there was no significant difference in change compared to the control group (*d* = –0.12), but there was also not a significant difference between the 90-min and 60-min conditions, *b* = –1.18, *SE* = 0.85, *p* = .17, *d* = –0.31. The 90-min condition also reported significantly greater reductions in parental hostility relative to the control group (*d* = –0.48; see Table 2 and Fig. 2d). Although reductions in parental hostility did not reach significance for the 60-min condition compared to the control group (*d* = –0.15), there was not a significant difference in change in hostility between the 90-min and 60-min conditions, *b* = –0.67, *SE* = 0.44, *p* = .13, *d* = –0.34. There were no significant differences in change in other dimensions of negative parenting, as well as parental psychological control, between any of the conditions (*p*s > .16, *d*s =|0.05 – 0.33|). As seen in Table S6, there were no significant differences observed for change in parental knowledge, parental hope, readiness for change or parental self-efficacy between any of the conditions (*p*s > .08, *d*s = 0.13—0.40).

**Table 2:** Effects of Intervention on Negative Parenting Behaviors

	**Random Intercept**				**Fixed Linear Time Slope**			
	*b*	*SE*	*p*	*d*	*b*	*SE*	*p*	*d*
**Negative Parenting**								
Intercept	**42.63**	**1.37**	** < .001**	**5.16**	––	––	––	
Time	––	––	––	––	–0.91	0.61	.14	–0.34
90-Min Condition	–1.36	2.06	.51	–0.11	–1.63	0.85	.06	–0.43
60-MinCondition	–0.10	2.24	.97	–0.01	–0.45	0.85	.60	–0.12
**Parental Hostility**								
Intercept	**18.13**	**0.67**	** < .001**	**4.38**	––	––	––	––
Time	––	––	––	––	–0.20	0.31	.52	–0.14
90-Min Condition	–0.06	1.01	.95	–0.01	**–0.96**	**0.44**	**.03**	**–0.48**
60-Min Condition	0.50	1.10	.65	0.08	–0.29	0.44	.51	–0.15
**Physical Control**								
Intercept	**7.19**	**0.48**	** < .001**	**2.85**	––	––	––	
Time	––	––	––	––	–0.22	0.14	.12	–0.39
90-Min Condition	0.49	0.73	.50	0.13	–0.15	0.20	.44	–0.19
60-Min Condition	–0.15	0.80	.85	–0.03	0.04	0.20	.83	0.05
**Lax Control**								
Intercept	**17.32**	**0.72**	** < .001**	**4.20**	––	––	––	––
Time	––	––	––	––	–0.42	0.28	.14	–0.35
90-Min Condition	–1.79	1.08	.10	–0.29	–0.51	0.40	.20	–0.30
60-Min Condition	–0.40	1.18	.73	–0.06	–0.32	0.40	.42	–0.19
**Psychological Control**								
Intercept	**12.96**	**0.66**	** < .001**	**3.37**	––	––	––	––
Time	––	––	––	––	–0.01	0.27	.96	–0.01
90-Min Condition	0.37	1.01	.71	0.06	–0.45	0.37	.23	–0.28
60-Min Condition	0.07	1.09	.95	0.01	–0.54	0.38	.16	–0.33

Waitlist control group is the reference. Bold estimates represent statistically significant paths (*p* < .05). *d* = partial Cohen’s *d*

### Post-Hoc Sensitivity Analyses Including Non-Attending Intervention Participants

Although primary intervention efficacy analyses were restricted to participants who attended at least one seminar, post-hoc sensitivity analyses were conducted to examine whether findings were consistent when all participants randomized to intervention conditions were considered, regardless of attendance. Baseline comparisons indicated no differences in outcome variables and only minor demographic differences related to employment status, number of caregivers present in the home, and geographic location (see Tables S7–S10). To evaluate the robustness of the efficacy findings, we conducted additional analyses including baseline data for participants who were assigned to an intervention condition but did not attend any seminars (missing outcome data were accounted for using REML in the multilevel models). Note that these participants had been contacted each time the seminars were offered across multiple rounds and were considered lost to follow-up due to their lack of participation. As shown in Tables S11–S13, results from the primary intervention outcome analyses remained largely consistent. The only difference was that parents in the 60-min condition demonstrated a marginal increase in parental knowledge relative to the control group (*p* = .05). However, this finding should be interpreted with caution, as it only emerged when including non-attending participants and was not found in the primary analyses.

## Discussion

Parents play a critical role in child development, influencing both emotional and behavioral outcomes (Morris et al., 2020). Given increasing concerns about child mental health (Racine et al., 2021), accessible parenting interventions are needed. The current study evaluated the feasibility, acceptability, and preliminary efficacy of Selected Triple P, delivered via telehealth, with the goal of expanding access to evidenced-based parenting support.

### Feasibility and Acceptability

Consistent with hypotheses, the current study provides the first known empirical support of Selected Triple P delivered through telehealth. Feasibility was demonstrated through multiple real-world indicators, including strong interest and enrollment (see CONSORT diagram), solid seminar attendance, and high levels of satisfaction with the intervention. Parents also reported strong satisfaction with the telehealth delivery, which aligns with previous work showing that virtual formats reduce barriers and broaden access (Butzner & Cuffee, 2021). Interestingly, acceptability ratings were slightly lower than satisfaction ratings. This suggests that, while parents found the intervention beneficial, some strategies may have felt less relevant, as the seminars were designed to offer a broad overview of parenting strategies for a wide age-range of children. Future work should explore tailoring content to family needs to enhance relevance. Additionally, although differences in satisfaction and acceptability ratings between the 90- and 60-min conditions were not statistically significant, the magnitude of these differences was moderate for some individual seminars, but not for overall satisfaction, suggesting this pattern may warrant closer examination in future studies with larger samples.

### Intervention Efficacy

Consistent with hypotheses, parents in the 90-min condition showed increases in overall positive parenting, which has been less explored in prior studies of Selected Triple P (Foskolos et al., 2023; Sanders et al., 2009; Sumargi et al., 2015). Specific improvements were also observed in positive reinforcement. Interestingly, there were not significant changes in parental warmth, support, or proactive parenting, perhaps due to a greater emphasis on praise throughout the seminars, making it more salient and therefore more likely for parents to implement (Sanders & Turner, 2012). These findings are in line with evidence suggesting that positive reinforcement is a crucial component of parenting interventions (Leijten et al., 2019).

Parents in the 90-min condition also showed significant reductions in hostility, with decreases in overall negative parenting behaviors approaching significance. This is in line with prior evidence that Selected Triple P reduces disruptive parenting (e.g., Foskolos et al., 2023). However, the intervention did not significantly impact physical control (i.e., corporal punishment) or lax control; this may be because parents’ current discipline strategies were not addressed in the seminars, and instead, focus was placed on introducing other strategies (e.g., time-out) that parents could utilize (Sanders & Turner, 2012). It’s possible that parents continued to use their current discipline strategies alongside these new strategies. Further, across conditions, parents reported low baseline levels of corporal punishment, which is consistent with research demonstrating lower rates of this behavior among families with a higher socioeconomic status (Hines et al., 2022) and may have limited the potential for observable change.

Similarly, parental psychological control remained unchanged, potentially due to a lack of direct intervention focus. It is important to note, however, that parents reported relatively low baseline levels of psychological control, which left little room for improvement. Given ample research demonstrating its harmful impacts on children (Hoeve et al., 2009), future work should explore whether targeted adaptations of this intervention could address this parenting behavior.

Contrary to hypotheses, and prior work (Cole & Molloy, 2023; Wittkowski et al., 2016), the intervention did not affect parental hope, readiness for change, or self-efficacy. This might indicate the presence of selection effects, such that parents who already felt more efficacious, hopeful, and ready to change were more likely to enroll in the study (Proctor et al., 2018; Thomas et al., 2022). Indeed, parents’ mean levels of these variables were high at baseline, leaving little room improvement. Further, some work suggests that self-efficacy can strengthen over time as parents engage in more effective parenting behaviors (Jones & Prinz, 2005). Therefore, it is possible that more time is needed post-intervention for such effects to emerge.

Further, the intervention did not lead to increases in parental knowledge. Previous research suggests that reducing ineffective parenting behaviors has a greater impact on child outcomes than knowledge gains alone (Winter et al., 2012). Further, knowing effective parenting strategies does not necessarily translate to their use in practice. Given that parenting interventions aim to improve child behavior, assessing actual parenting practices may be more informative than measuring knowledge in hypothetical scenarios. Nonetheless, parental knowledge may still facilitate parent behavior change (Winter et al., 2012), so more work is needed to clarify its role in improving child outcomes.

Across all outcomes, patterns of effect sizes suggested small, positive effects in the expected direction—even when not statistically significant. These subtle shifts in parenting behaviors highlight the possibility that a larger sample with greater power may detect more intervention effects. Accordingly, a post-hoc power analysis confirmed that the current sample was underpowered to detect small effects, underscoring the importance of replication with a larger trial. As a robustness check, we also conducted supplemental sensitivity analyses that included baseline data for participants assigned to an intervention condition but did not attend any seminars. Importantly, findings remained largely consistent with the primary results. One marginal finding emerged for the 60-min condition on parental knowledge, though this was not observed in the primary analyses and should be interpreted with caution.

It is also important to note that parents were included in efficacy analyses after attending just one seminar, resulting in variability in intervention dosage within the analytic sample. Among parents included in the efficacy analyses, the majority attended all three seminars (68.8% in the 90-min condition; 79.2% in the 60-min condition). Nonetheless, a subset attended only one or two seminars, indicating heterogeneity in the amount of intervention exposure. This variability may have influenced the magnitude of observed changes and limits conclusions regarding dose–response effects. Overall, these results increase confidence in the observed patterns while underscoring the need for future studies with larger samples to clarify the magnitude and mechanisms of change in brief, universal parenting interventions.

### Comparisons Between 90-Minute and 60-Minute Conditions

Interestingly, although there were improvements in the expected direction (see Fig. 2), there were no significant changes across intervention outcomes for parents in the 60-min condition when compared to the waitlist control group. However, outcome differences between the 90-min and 60-min conditions were small, indicating that parents in the 60-min condition improved in ways that were not statistically distinguishable from those in the 90-min condition. These patterns suggest that a more concise intervention format may offer meaningful benefits to parents. While this study was powered to detect medium to large effects, many of the observed differences between intervention conditions were small, raising the possibility that a larger sample size may be needed to detect significant differences 6. Importantly, from a public health perspective, the potential utility of a 60-min, possibly pre-recorded, three-session intervention format has meaningful implications for scalability and dissemination, particularly in communities where access to mental health providers is limited. More work is needed to clarify the role of session length and active discussion in enhancing outcomes.

### Implications

Findings from this study support the feasibility, acceptability, and preliminary efficacy of a brief universal parenting intervention delivered via telehealth. Strong attendance rates and high satisfaction with the telehealth format suggest that virtual delivery is well received by parents and may reduce common participation barriers, such as access to transportation and childcare (Baker et al., 2011). Notably, most participants lived in a city outside of where the research team was located, indicating that telehealth broadened access to families who may not have been able to attend in-person. Importantly, parents also demonstrated improvements in parenting behaviors, indicating that the intervention was not only accessible, but it was also efficacious.

These findings also support the utility of brief, scalable interventions. While traditional parenting interventions often target families with clinically significant behavior problems and require intensive resources (Salari & Enebrink, 2018), brief, universal interventions can reach more families with fewer demands on providers. Such interventions may also play a role in prevention, such that the promotion of positive parenting may, in turn, prevent the development of more severe problems that require more intensive interventions (Salari & Enebrink, 2018). Given their brief format and minimal resource demands, these interventions could also be feasibly implemented at scale through public systems, such as statewide school initiatives, as a part of broader prevention efforts. Notably, variability in session attendance in the current study reflects real-world participation patterns, further highlighting the potential value of flexible intervention models that can still benefit families even when full participation is not feasible.

Finally, findings raise questions about the role of active discussion in parenting interventions. While prior work emphasizes the importance of actively practicing skills with a mental health provider (Morris et al., 2020), results from this study suggest that a discussion component may not be necessary for satisfaction or engagement. Further, as previously noted, effect sizes for the differences between the 60- and 90-min conditions were small. Given the greater scalability of interventions that do not require a mental health provider for delivery, it is possible that the added benefit from the active discussion component may be outweighed by the advantage of reaching a larger number of families. However, more clinical trials with larger sample sizes are needed to further explore the potential benefits of this approach.

### Strengths, Limitations, and Future Directions

The current study has several strengths, including the use of a randomized controlled trial design. Notably, there were no significant baseline differences between conditions, indicating that randomization was successful. The current study also included a geographically diverse sample, with most participants providing a non-local address, capturing both urban and rural living environments. This demonstrates the feasibility of delivering parenting interventions across regions and reaching families who might otherwise lack access. High intervention fidelity and relatively strong retention rates further enhance the internal validity.

However, several limitations should be acknowledged. First, although geographically diverse, the sample was primarily composed of White, highly-educated parents with higher socioeconomic status, which may limit generalizability. Additionally, selection effects may have influenced the sample, such that parents who were already motivated to improve their parenting may have been more likely to enroll. This is particularly important given that parents who initiated outside mental health services during the study were excluded from analyses (see Online Supplementary Material), possibly resulting in a sample with fewer baseline challenges. Although random assignment supports internal validity, future research should prioritize broadening the sample to include more diverse parents.

Second, outcomes were assessed solely through parent-reports 2 months post-intervention, limiting conclusions about the durability of observed changes or their impact on child functioning. Although positive shifts in parenting behaviors emerged, it remains unclear whether these gains are maintained over time or translate to improvements in child outcomes—arguably the ultimate target of parenting interventions (Morris et al., 2020). Additionally, parents’ awareness of effective strategies may have influenced their self-reports, potentially inflating perceived changes in behaviors. Future research should address these limitations by incorporating longer-term follow-up assessments and integrating multi-informant approaches (e.g., observations) to evaluate both parenting behaviors and downstream effects on child functioning.

A third limitation was that parents were included in the efficacy analyses if they attended at least one seminar, but it did not matter which seminar they attended. Because each seminar targeted different parenting domains, and participants received differing amounts of intervention exposure, it is not possible to determine which content contributed most to changes in parenting behavior. Future studies should use dismantling designs to identify what seminar content is most strongly associated with changes in outcomes.

Fourth, although follow-up data were only available for parents who attended at least one seminar, we conducted supplemental sensitivity analyses using baseline data from parents who were randomized to an intervention condition but did not attend any seminars. These analyses yielded consistent results, with only one additional marginally significant effect observed. Still, the absence of post-intervention data from this group limits the ability to draw conclusions about the full intent-to-treat sample. Innovative strategies to assess or retain participants who do not initiate the intervention are warranted in future trials.

An additional consideration relates to potential systematic differences between parents assigned to an intervention condition who attended at least one seminar and those who did not attend any seminars. Although baseline outcome variables did not differ between these groups, parents who attended at least one seminar were more likely to be unemployed and to report having a second caregiver in the home, whereas non-attenders were more likely to reside outside of the city in which the research team was located (see Online Supplementary Material). These patterns suggest that participation may have been influenced by differences in time availability, caregiving support, or other logistical factors. Although seminars were offered on multiple days and at varied times, parents were still required to attend seminars at a scheduled time. As such, findings may reflect families with greater scheduling flexibility or support, and caution is warranted when generalizing results to parents facing greater structural barriers to participation. Although telehealth delivery was intended to reduce common logistical barriers to participation, continued work is needed to further increase accessibility, including exploring alternative dissemination strategies (e.g., asynchronous or self-paced formats) that allow parents to access parenting support without requiring attendance at specific times.

Finally, a post-hoc power analysis indicated that the current study was underpowered to detect small effects. Nonetheless, the majority of effects were in the expected direction, including small but consistent improvements in parenting behaviors, parental knowledge, hope, readiness for change, and self-efficacy. This pattern underscores the potential promise of the intervention and suggests that a larger clinical trial may be necessary to fully detect and replicate these effects. Notably, over 500 parents expressed interest in the study but did not meet eligibility criteria, highlighting the potential for scalability and the appeal of this brief parenting model.

## Conclusion

Brief, universal parenting interventions may help narrow the gap between families in need of mental health services and those able to access them. Findings provide evidence that a brief positive parenting seminar series delivered via telehealth is both feasible and acceptable for parents of children ages 2–12 years old across a broad geographic region. Parents in the intervention condition with an active discussion component showed significant improvements in parenting behaviors, including reduced hostility and overall negative parenting and increased positive reinforcement and overall positive parenting. Although improvements in the no-discussion condition were not statistically significant, there were also no significant differences between the two intervention groups, suggesting that active discussion may not be essential for program effectiveness. Together, these findings have important implications for expanding access to evidence-based parenting support. Interventions that do not rely on mental health providers for delivery could expand access to mental healthcare, particularly in underserved or rural areas. Overall, this study highlights the value of innovative, scalable approaches to mental health service delivery.

## Supplementary Information

Below is the link to the electronic supplementary material.

ESM1 (DOCX 110 KB)

## Data Availability

The research data are available from the corresponding author upon request.

## Footnotes

1 Many adults can provide caregiving duties. The term parents is used throughout the manuscript but refers to any adult that provides regular caregiving duties.

2 See online supplementary material for baseline comparisons between parents who were assigned to an intervention condition and attended at least one seminar and those who did not.

3 Two independent raters (i.e., not seminar co-leaders) watched the recordings and coded for fidelity using checklists developed by Triple P. Interrater reliability was excellent (κ = 1.0). Across all seminars in both conditions, all seminar content (100%) was covered.

4 The items that were used can be found in the online supplementary material.

5 Information about missing data can be found in the online supplementary material. There was a 91.8% response rate for the 2-month follow-up assessment across all conditions, with no significant difference in missing data across conditions, χ^2 ^(2, 97) = 2.50, *p* = .29.

6 A post-hoc power analysis using the observed effect sizes (*f* = 0.03 to *f* = 0.17) for differences between intervention conditions indicated that detecting smaller effects would have required a substantially larger sample size (i.e., n ≥337).
