# Supplementary material for: Evaluating A Brief Telehealth Positive Parenting Intervention: A Randomized Controlled Trial
Source: Prev Sci. 2026 Mar 21;27(3):390–405. doi: 10.1007/s11121-026-01894-3 (PMC13102863; doi:10.1007/s11121-026-01894-3)
Supplement: Supplementary file 1 — (DOCX 110 KB) [file 11121_2026_1894_MOESM1_ESM.docx]

**SUPPLEMENTAL MATERIAL**

### **Method**

**Measures**

***Items Included in the Modified TARF-R Acceptability Measure***

The following 9-items were selected from the Treatment Acceptability Rating Form-Revised (TARF-R; Reimers et al., 1992) for the current study. Items were chosen to avoid overlap with other measures and to ensure relevance to the intervention context. Parents were asked to respond to each item on a 7-point scale ranging from 1 (*Not at all*) to 7 (*Very much)*.

1. How much did you like the procedures discussed in the seminar?
2. How acceptable did you find the procedures discussed in the seminar?
3. How likely are these strategies to make permanent improvements in your child’s behavior?
4. How likely are these strategies going to be effective for your child?
5. How confident are you that the strategies in the seminar are effective?
6. To what extent do you think there might be disadvantages to following the strategies presented in the seminar?
7. How disruptive will it be to the family in general to carry out the strategies presented in this seminar?
8. How time consuming will it be to carry out the strategies presented in this seminar?
9. To what extent will carrying out the strategies presented in this seminar fit into the family routine?

### **Results**

### **Missing Data**

At baseline, only one participant had missing outcome data on one variable. There was no other missing outcome data at baseline. At the 2-month follow-up, 27 parents assigned to the 90-minute condition (84.4% response rate), 23 parents assigned to the 60-minute condition (95.8% response rate), and 39 parents assigned to the waitlist control group (95.1% response rate) completed the survey (91.8% overall response rate). A chi-square test indicated no significant differences in missing data across conditions χ^2^ (2, 97) = 2.50, *p* = .29.

At the 2-month follow-up, data were excluded for 15 parents because they indicated that they (90-minute condition: *n* = 1, 60-minute condition: *n* = 2, waitlist control condition: *n* = 2) or their child (90-minute condition: *n* = 3, 60-minute condition: *n* = 1, waitlist control condition: *n* = 6) began mental health treatment since completing the baseline survey; however, these parents’ baseline data were retained, and they were included in the final analysis. Three parents appeared to report on different children at the 2-month follow-up assessment (90-minute condition: *n* = 2, waitlist control condition: *n* = 1), so their data on outcomes referring to their parenting towards a specific child (i.e., positive/negative parenting) were excluded, but their data on outcomes related to parenting more broadly (e.g., parental hope) were retained.

**Comparison of Intervention Participants Who Did and Did Not Attend Any Seminars Following Randomization**

***Differences in Demographic Variables and Baseline Differences in Outcome Variables***

Although parents assigned to an intervention condition were contacted multiple times with reminders and invitations to attend the seminars each time they were offered, a subset did not attend any seminars. These parents were considered lost to follow-up given that they received no dose of the intervention. To evaluate the representativeness of the analytic sample, we compared demographic characteristics and baseline levels of outcome variables between parents who were randomized to an intervention condition but did not attend any seminars (*n* = 23) and those who attended at least one seminar (*n* = 56).

As presented in Tables S7-S9, the majority of demographic characteristics did not differ significantly between groups. However, parents who attended at least one seminar were marginally more likely to be unemployed (*p* = .05) and to report having a second caregiver in the home (*p* = .05), whereas parents who did not attend any seminars were significantly more likely to reside outside the city in which the research team was located (*p* = .002).

Table S10 presents baseline comparisons in outcome variables between these two groups. There were no significant differences on any variable between parents assigned to an intervention condition who attended no seminars and those who attended at least one seminar.

***Exploratory Analyses Including Baseline Data from Parents Who Did Not Attend Seminars***

To evaluate the robustness of the intervention findings, we re-ran the primary efficacy analyses including the baseline data of parents who were randomly assigned to an intervention condition but did not attend any seminars. As seen in Tables S11-S13, the pattern of results remained largely consistent, such that parents in the 90-minute condition continued to report significant increases in positive parenting and positive reinforcement along with reductions in hostility and negative parenting compared to the control group.

One new finding that emerged in these analyses was a marginally significant increase in parental knowledge among parents in the 60-minute condition compared to the control group (*p* = .05; see Table S13). Note, however, that this effect was not observed in the original analyses excluding parents who did not attend any seminars, so it should be interpreted cautiously. Taken together, the overall pattern of findings was stable and suggests that intervention effects are robust.

Consistent with the original analyses, there were no significant differences between the 90- and 60-minute conditions on any outcome in the exploratory analyses that included parents who were randomized to an intervention condition but did not attend any seminars. Specifically, there were no significant differences in change in positive parenting (*p* = .63, *d* = 0.11), negative parenting (*p* = .27, *d* = -0.24), positive reinforcement (*p* = .18, *d* = 0.29), warmth (*p* = .73, *d* = 0.08), support (*p* = .47, *d* = 0.16), proactive parenting (*p* = .60, *d* = -0.11), hostility (*p* = .12, *d* = -0.32), physical control (*p* = .53, *d* = -0.15), lax control (*p* = .73, *d* = -0.07), psychological control (*p* = .55, *d* = 0.14), parental knowledge (*p* = .24, *d* = -0.26), hope (*p* = .66, *d* = -0.10), readiness for change (*p* = .41, *d* = -0.19), or parental self-efficacy (*p* = .81, *d* = 0.04).

# **Table S1**

*Demographic Characteristics of Parents by Condition*

| Variable | 90-Minute | | 60-Minute | | Waitlist Control | |  |  |  |
| --- | --- | --- | --- | --- | --- | --- | --- | --- | --- |
|  | ***M*** | ***SD*** | ***M*** | ***SD*** | ***M*** | ***SD*** | ***F*** | ***p*** | ***η^2^*** |
| Parent age (years) | 36.42 | 7.10 | 35.04 | 5.67 | 35.37 | 6.57 | 0.35 | .703 | .01 |
|  | ***n*** | **%** | ***n*** | **%** | ***n*** | **%** | ***χ^2^*** | ***p*** | ***V*** |
| Parent Gender |  |  |  |  |  |  | 1.38 | .50 | .12 |
| Male | 6 | 18.8 | 3 | 12.5 | 10 | 24.4 |  |  |  |
| Female | 26 | 81.3 | 21 | 87.5 | 31 | 75.6 |  |  |  |
| Parent Ethnicity |  |  |  |  |  |  | 4.08 | .13 | .21 |
| Hispanic/Latine | 5 | 15.6 | 6 | 25.0 | 15 | 36.6 |  |  |  |
| Not Hispanic/Latine | 27 | 84.4 | 16 | 75.0 | 26 | 63.4 |  |  |  |
| Parent Race |  |  |  |  |  |  | 6.85 | .55 | .19 |
| White | 24 | 75.0 | 17 | 73.9 | 28 | 68.3 |  |  |  |
| Black | 5 | 15.6 | 2 | 8.7 | 8 | 19.5 |  |  |  |
| Asian | 2 | 6.3 | 0 | 0.0 | 1 | 2.4 |  |  |  |
| American Indian or Alaska Native | 0 | 0.0 | 1 | 4.3 | 2 | 4.9 |  |  |  |
| Other | 1 | 3.1 | 3 | 13.0 | 2 | 4.9 |  |  |  |
| Parent Relationship to Child |  |  |  |  |  |  | 7.43 | .69 | .20 |
| Mother | 21 | 65.6 | 21 | 87.5 | 28 | 68.3 |  |  |  |
| Father | 6 | 18.8 | 3 | 12.5 | 10 | 24.4 |  |  |  |
| Stepmother | 1 | 3.1 | 0 | 0.0 | 1 | 2.4 |  |  |  |
| Adoptive Mother | 2 | 6.3 | 0 | 0.0 | 1 | 2.4 |  |  |  |
| Grandmother | 1 | 3.1 | 0 | 0.0 | 1 | 2.4 |  |  |  |
| Aunt | 1 | 3.1 | 0 | 0.0 | 0 | 0.0 |  |  |  |

*Note.* 90-Minute Condition (*n* = 32). 60-Minute Condition (*n* = 24). Waitlist Control Condition (*n* = 41). V = Cramér’s V.

**Table S1** (continued).

| Variable | 90-Minute | | 60-Minute | | Waitlist Control | |  |  |  |
| --- | --- | --- | --- | --- | --- | --- | --- | --- | --- |
|  | ***n*** | **%** | ***n*** | **%** | ***n*** | **%** | ***χ^2^*** | ***p*** | ***V*** |
| Marital Status |  |  |  |  |  |  | 7.22 | .84 | .19 |
| Married – living together | 26 | 81.3 | 18 | 75.0 | 29 | 70.7 |  |  |  |
| Living with Partner | 2 | 6.3 | 2 | 8.3 | 2 | 4.9 |  |  |  |
| Married – not living together | 0 | 0.0 | 1 | 4.2 | 2 | 4.9 |  |  |  |
| Divorced | 3 | 9.4 | 2 | 8.3 | 5 | 12.2 |  |  |  |
| Legally Separated | 0 | 0.0 | 0 | 0.0 | 1 | 2.4 |  |  |  |
| Widowed | 1 | 3.1 | 0 | 0.0 | 0 | 0.0 |  |  |  |
| Never married, not living with partner | 0 | 0.0 | 1 | 4.2 | 2 | 4.9 |  |  |  |
| Parent Education Level |  |  |  |  |  |  | 13.23 | .66 | .23 |
| High school graduate | 1 | 3.1 | 2 | 8.3 | 4 | 9.8 |  |  |  |
| Trade school or technical school | 3 | 9.4 | 0 | 0.0 | 1 | 2.4 |  |  |  |
| Some college | 9 | 28.1 | 4 | 16.7 | 11 | 26.8 |  |  |  |
| Bachelor’s degree | 8 | 25.0 | 9 | 37.5 | 9 | 22.0 |  |  |  |
| Master’s degree | 7 | 21.9 | 8 | 33.3 | 9 | 22.0 |  |  |  |
| Professional degree (e.g., MD, DDS, JD) | 2 | 6.3 | 0 | 0.0 | 2 | 4.9 |  |  |  |
| Doctorate degree (e.g., Ph.D., Ed.D.) | 2 | 6.3 | 1 | 4.2 | 5 | 12.2 |  |  |  |
| Parent Employment |  |  |  |  |  |  | 2.45 | .88 | .11 |
| Full-time employment | 21 | 65.6 | 15 | 62.5 | 28 | 68.3 |  |  |  |
| Part-time employment | 2 | 6.3 | 3 | 12.5 | 3 | 7.3 |  |  |  |
| Unemployed | 9 | 28.1 | 6 | 25.0 | 9 | 22.0 |  |  |  |
| Retired | 0 | 0.0 | 0 | 0.0 | 1 | 2.4 |  |  |  |

*Note.* 90-Minute Condition (*n* = 32). 60-Minute Condition (*n* = 24). Waitlist Control Condition (*n* = 41). V = Cramér’s V.

# **Table S2**

*Demographic Characteristics of Children by Condition*

| Variable | 90-Minute | | 60-Minute | | Waitlist Control | |  | |  | |
| --- | --- | --- | --- | --- | --- | --- | --- | --- | --- | --- |
|  | ***M*** | ***SD*** | ***M*** | ***SD*** | ***M*** | ***SD*** | ***F*** | ***p*** | | ***η^2^*** |
| Child age (years) | 6.59 | 3.35 | 6.42 | 3.28 | 6.71 | 3.12 | 0.06 | .941 | | .00 |
|  | ***n*** | **%** | ***n*** | **%** | ***n*** | **%** | ***χ^2^*** | ***p*** | | ***V*** |
| Child Gender |  |  |  |  |  |  | 5.05 | .08 | | .23 |
| Male | 24 | 75.0 | 11 | 45.8 | 24 | 58.5 |  |  | |  |
| Female | 8 | 25.0 | 13 | 54.2 | 17 | 41.5 |  |  | |  |
| Child Ethnicity |  |  |  |  |  |  | 1.43 | .49 | | .12 |
| Hispanic/Latine | 10 | 31.3 | 8 | 33.3 | 18 | 43.9 |  |  | |  |
| Not Hispanic/Latine | 22 | 68.8 | 16 | 66.7 | 23 | 56.1 |  |  | |  |
| Child Race |  |  |  |  |  |  | 3.96 | .95 | | .14 |
| White | 22 | 68.8 | 16 | 69.6 | 27 | 65.9 |  |  | |  |
| Black | 5 | 15.6 | 3 | 13.0 | 8 | 19.5 |  |  | |  |
| Asian | 2 | 6.3 | 0 | 0.0 | 1 | 2.4 |  |  | |  |
| American Indian or Alaska Native | 1 | 3.1 | 1 | 4.3 | 2 | 4.9 |  |  | |  |
| More than one race | 0 | 0.0 | 1 | 4.3 | 1 | 2.4 |  |  | |  |
| Other | 2 | 6.3 | 2 | 8.7 | 2 | 4.9 |  |  | |  |

*Note.* 90-Minute Condition (*n* = 32). 60-Minute Condition (*n* = 24). Waitlist Control Condition (*n* = 41). V = Cramér’s V.

# **Table S3**

*Household Characteristics by Condition*

| Variable | 90-Minute | | 60-Minute | | Waitlist Control | |  |  |  |
| --- | --- | --- | --- | --- | --- | --- | --- | --- | --- |
|  | ***M*** | ***SD*** | ***M*** | ***SD*** | ***M*** | ***SD*** | ***F*** | ***p*** | ***η^2^*** |
| Number of children in the household | 1.97 | 1.12 | 2.21 | 0.83 | 1.88 | 1.12 | 0.75 | .48 | .02 |
| Number of adults in the household | 1.94 | 0.35 | 1.83 | 0.48 | 1.90 | 0.54 | 0.34 | .71 | .01 |
| Household income | $124,200 | $110,848 | $78,989 | $54,976 | $93,3449 | $60,746 | 2.30 | .11 | .05 |
|  | ***n*** | **%** | ***n*** | **%** | ***n*** | **%** | ***χ^2^*** | ***p*** | ***V*** |
| Other Child Caregiver in the home |  |  |  |  |  |  | 0.01 | .99 | .01 |
| Yes | 24 | 75.0 | 18 | 75.0 | 31 | 75.6 |  |  |  |
| No | 8 | 25.0 | 6 | 25.0 | 10 | 24.4 |  |  |  |
| Local Address |  |  |  |  |  |  | 2.82 | .24 | .17 |
| Yes | 9 | 28.1 | 12 | 50.0 | 15 | 36.6 |  |  |  |
| No | 23 | 71.9 | 12 | 50.0 | 26 | 63.4 |  |  |  |
| Rural Address |  |  |  |  |  |  | 0.04 | .98 | .02 |
| Yes | 4 | 16.0 | 4 | 16.7 | 5 | 14.7 |  |  |  |
| No | 21 | 84.0 | 20 | 83.3 | 29 | 85.3 |  |  |  |

*Note.* 90-Minute Condition (*n* = 32). 60-Minute Condition (*n* = 24). Waitlist Control Condition (*n* = 41). V = Cramér’s V. Local address incudes all parents who listed a mailing address in the city in which the research team was located.

Rural address includes participants who provided a street address and zip code that was considered to be rural according to the Health Resources & Services Administration.

# **Table S4**

*Baseline Differences in Intervention Outcomes by Condition*

| Variable | 90-Minute | | 60-Minute | | Waitlist Control | |  | |  |
| --- | --- | --- | --- | --- | --- | --- | --- | --- | --- |
|  | ***M*** | ***SD*** | ***M*** | ***SD*** | ***M*** | ***SD*** | ***F*** | ***p*** | ***η^2^*** |
| Positive Parenting | 66.25 | 6.57 | 67.50 | 4.26 | 67.20 | 6.51 | 0.35 | .71 | .01 |
| Parental Warmth | 12.72 | 1.73 | 13.58 | 1.32 | 12.88 | 1.79 | 2.04 | .14 | .04 |
| Parental Support | 12.91 | 1.79 | 12.75 | 1.19 | 13.00 | 1.55 | 0.20 | .83 | .00 |
| Positive Reinforcement | 16.88 | 2.43 | 17.21 | 1.19 | 17.29 | 2.17 | 0.36 | .70 | .01 |
| Proactive Parenting | 23.75 | 2.62 | 23.96 | 2.29 | 24.02 | 2.74 | 0.10 | .90 | .00 |
| Negative Parenting | 41.28 | 9.45 | 42.72 | 10.26 | 42.56 | 9.07 | 0.22 | .81 | .01 |
| Parental Hostility | 18.09 | 4.55 | 18.71 | 4.50 | 18.05 | 4.60 | 0.18 | .84 | .00 |
| Physical Control | 7.72 | 3.20 | 6.96 | 3.61 | 7.20 | 3.02 | 0.42 | .66 | .01 |
| Lax Control | 15.47 | 4.91 | 17.00 | 5.62 | 17.32 | 4.77 | 1.30 | .28 | .03 |
| Parental Psychological Control | 13.35 | 5.28 | 13.13 | 4.74 | 12.98 | 4.10 | 0.06 | .94 | .00 |
| Parental Knowledge (%) | 49.22 | 14.41 | 49.79 | 15.60 | 45.73 | 14.35 | 0.77 | .46 | .02 |
| Parental Hope | 35.32 | 4.88 | 35.42 | 2.98 | 35.76 | 3.58 | 0.13 | .88 | .00 |
| Readiness for Change | 27.34 | 3.16 | 26.96 | 3.38 | 26.93 | 3.45 | 0.16 | .85 | .00 |
| Parental Self-Efficacy | 19.75 | 4.42 | 20.21 | 2.32 | 20.44 | 2.48 | 0.42 | .66 | .01 |

*Note.* 90-Minute Condition (*n* = 32). 60-Minute Condition (*n* = 24). Waitlist Control Condition (*n* = 41). Parental knowledge is measured as the percentage of the items that parents got correct.

# **Table S5**

*Differences in Satisfaction and Acceptability Between Intervention Conditions*

| Variable | 90-Minute | | 60-Minute | |  | |  |
| --- | --- | --- | --- | --- | --- | --- | --- |
|  | ***M*** | ***SD*** | ***M*** | ***SD*** | ***t*** | ***p*** | ***d*** |
| **Post-Seminar Satisfaction** |  |  |  |  |  |  |  |
| Seminar 1 Satisfaction | 6.50 | 0.55 | 6.31 | 0.54 | 1.17 | .12 | 0.34 |
| Seminar 2 Satisfaction | 6.58 | 0.50 | 6.34 | 0.65 | 1.32 | .10 | 0.42 |
| Seminar 3 Satisfaction | 6.45 | 0.78 | 6.45 | 0.59 | 0.03 | .49 | 0.01 |
| Total Average Satisfaction | 6.51 | 0.60 | 6.40 | 0.53 | 0.67 | .25 | 0.19 |
| **Satisfaction with Virtual Modality** | 6.89 | 0.27 | 6.73 | 0.66 | 1.04 | .15 | 0.34 |
| **Global Satisfaction with Intervention** | 3.58 | 0.42 | 3.47 | 0.39 | 0.81 | .21 | 0.25 |
| **Acceptability** |  |  |  |  |  |  |  |
| Seminar 1 Acceptability | 5.80 | .70 | 5.69 | 0.66 | 0.54 | .30 | 0.16 |
| Seminar 2 Acceptability | 5.91 | 0.77 | 5.64 | 0.73 | 1.12 | .14 | 0.36 |
| Seminar 3 Acceptability | 5.83 | 0.70 | 5.72 | 0.69 | 0.54 | .30 | 0.16 |
| Total Average Acceptability | 5.84 | 0.64 | 5.69 | 0.63 | 0.89 | .19 | 0.25 |

*Note*. Post-seminar satisfaction was rated on a 7-point scale, with higher scores indicating greater satisfaction. Seminar 1 satisfaction (*n* = 50). Seminar 2 satisfaction (*n* = 40). Seminar 3 satisfaction (*n* = 43). Average satisfaction (*n* = 52). Satisfaction with virtual modality response scale ranged from 1-7. Satisfaction with virtual modality (*n* = 38). Global satisfaction was rated on a 4-point scale, with higher scores indicating greater satisfaction. Acceptability was rated on a 7-point scale, with higher scores indicating greater acceptability. Seminar 1 acceptability (*n* = 50). Seminar 2 acceptability (*n* = 40). Seminar 3 acceptability (*n* = 43). Average acceptability (*n* = 52). d = Cohen’s d.

# **Table S6**

*Effects of Intervention on Parental Knowledge, Parental Hope, Readiness for Change, and Parental Self-Efficacy*

|  | **Random Intercept** | | | | **Fixed Linear Time Slope** | | | |
| --- | --- | --- | --- | --- | --- | --- | --- | --- |
|  | *b* | *SE* | *p* | *d* | *b* | *SE* | *p* | *d* |
| **Parental Knowledge** |  |  |  |  |  |  |  |  |
| Intercept | **0.46** | **0.02** | **< .001** | **3.41** | –– | –– | –– | –– |
| Time | –– | –– | –– | –– | 0.01 | 0.01 | .15 | 0.33 |
| 90-Minute Condition | 0.04 | 0.04 | .31 | 0.18 | 0.01 | 0.01 | .47 | 0.16 |
| 60-Minute Condition | 0.05 | 0.04 | .25 | 0.21 | 0.02 | 0.01 | .08 | 0.40 |
| **Parental Hope** |  |  |  |  |  |  |  |  |
| Intercept | **35.78** | **0.56** | **< .001** | **11.18** | –– | –– | –– |  |
| Time | –– | –– | –– | –– | 0.12 | 0.22 | .58 | 0.14 |
| 90-Minute Condition | –0.45 | 0.84 | .59 | –0.09 | 0.47 | 0.31 | .13 | 0.37 |
| 60-Minute Condition | –0.33 | 0.91 | .72 | –0.06 | 0.43 | 0.31 | .17 | 0.34 |
| **Readiness for Change** |  |  |  |  |  |  |  |  |
| Intercept | **26.92** | **0.52** | **< .001** | **9.00** | –– | –– | –– | –– |
| Time | –– | –– | –– | –– | –0.14 | 0.20 | .50 | –0.16 |
| 90-Minute Condition | 0.48 | 0.78 | .54 | 0.11 | 0.16 | 0.28 | .56 | 0.13 |
| 60-Minute Condition | 0.01 | 0.85 | .99 | 0.00 | 0.36 | 0.29 | .21 | 0.29 |
| **Parental Self-Efficacy** |  |  |  |  |  |  |  |  |
| Intercept | **20.47** | **0.46** | **< .001** | **8.11** | –– | –– | –– | –– |
| Time | –– | –– | –– | –– | 0.29 | 0.17 | .10 | 0.44 |
| 90-Minute Condition | –0.63 | 0.70 | .37 | –0.17 | 0.31 | 0.24 | .20 | 0.33 |
| 60-Minute Condition | –0.19 | 0.76 | .80 | –0.05 | 0.19 | 0.25 | .44 | 0.20 |

*Note.* Waitlist control group is the reference. Bold estimates represent statistically significant paths (*p* < .05). *d* = partial Cohen’s *d*.

**Table S7**

*Parent Demographic Characteristics by Seminar Attendance Status (Intervention Groups Only)*

| Variable | Attended  0 Seminars | | Attended  ≥1 Seminar | |  |  |  |
| --- | --- | --- | --- | --- | --- | --- | --- |
|  | ***M*** | ***SD*** | ***M*** | ***SD*** | ***t*** | ***p*** | ***d*** |
| Parent age (years) | 36.00 | 9.83 | 35.32 | 7.43 | 0.33 | .74 | .08 |
|  | ***n*** | **%** | ***n*** | **%** | ***χ^2^*** | ***p*** | ***V*** |
| Parent Gender |  |  |  |  | 1.06 | .30 | .12 |
| Male | 6 | 26.1 | 9 | 16.1 |  |  |  |
| Female | 17 | 73.9 | 47 | 83.9 |  |  |  |
| Parent Ethnicity |  |  |  |  | 1.42 | .23 | .13 |
| Hispanic/Latine | 21 | 91.3 | 45 | 80.4 |  |  |  |
| Not Hispanic/Latine | 2 | 8.7 | 11 | 19.6 |  |  |  |
| Parent Race |  |  |  |  | 2.37 | .67 | .17 |
| White | 16 | 69.6 | 41 | 74.5 |  |  |  |
| Black | 5 | 21.7 | 7 | 12.7 |  |  |  |
| Asian | 0 | 0.0 | 2 | 3.6 |  |  |  |
| American Indian or Alaska Native | 1 | 4.3 | 1 | 1.8 |  |  |  |
| Other | 1 | 4.3 | 4 | 7.3 |  |  |  |
| Parent Relationship to Child |  |  |  |  | 7.70 | .36 | .31 |
| Mother | 14 | 60.9 | 42 | 75.9 |  |  |  |
| Father | 5 | 21.7 | 9 | 16.1 |  |  |  |
| Stepmother | 0 | 0.0 | 1 | 1.8 |  |  |  |
| Adoptive Mother | 2 | 8.7 | 2 | 3.6 |  |  |  |
| Grandmother | 0 | 0.0 | 1 | 1.8 |  |  |  |
| Aunt | 0 | 0.0 | 1 | 1.8 |  |  |  |
| Other | 2 | 8.7 | 0 | 0.0 |  |  |  |

*Note.* Attended 0 Seminars (*n* = 23). Attended ≥1 Seminar (*n* = 56). *d* = Cohen’s d. V = Cramér’s V.

**Table S7** (continued).

| Variable | Attended  0 Seminars | | Attended  ≥1 Seminar | |  |  |  |
| --- | --- | --- | --- | --- | --- | --- | --- |
|  | ***n*** | **%** | ***n*** | **%** | ***χ^2^*** | ***p*** | ***V*** |
| Marital Status |  |  |  |  | 5.36 | .37 | .26 |
| Married – living together | 15 | 65.2 | 44 | 78.6 |  |  |  |
| Living with Partner | 2 | 8.7 | 4 | 7.1 |  |  |  |
| Married – not living together | 1 | 4.3 | 1 | 1.8 |  |  |  |
| Divorced | 2 | 8.7 | 5 | 8.9 |  |  |  |
| Legally Separated | 0 | 0.0 | 0 | 0.0 |  |  |  |
| Widowed | 0 | 0.0 | 1 | 0.0 |  |  |  |
| Never married, not living with partner | 3 | 13.0 | 1 | 1.8 |  |  |  |
| Parent Education Level |  |  |  |  | 5.87 | .44 | .27 |
| High school graduate | 4 | 17.4 | 3 | 5.4 |  |  |  |
| Trade school or technical school | 0 | 0.0 | 3 | 5.4 |  |  |  |
| Some college | 3 | 13.0 | 13 | 23.2 |  |  |  |
| Bachelor’s degree | 8 | 34.8 | 17 | 30.4 |  |  |  |
| Master’s degree | 5 | 21.7 | 15 | 26.8 |  |  |  |
| Professional degree (e.g., MD, DDS, JD) | 3 | 13.0 | 2 | 3.4 |  |  |  |
| Doctorate degree (e.g., Ph.D., Ed.D.) | 1 | 4.3 | 3 | 5.4 |  |  |  |
| Parent Employment |  |  |  |  | 6.15 | .05 | .28 |
| Full-time employment | 21 | 91.3 | 36 | 62.3 |  |  |  |
| Part-time employment | 1 | 4.3 | 5 | 8.9 |  |  |  |
| Unemployed | 1 | 4.3 | 15 | 26.8 |  |  |  |
| Retired | 0 | 0.0 | 0 | 0.0 |  |  |  |

*Note.* Attended 0 Seminars (*n* = 23). Attended ≥1 Seminar (*n* = 56). V = Cramér’s V.

# **Table S8**

*Child Demographic Characteristics by Seminar Attendance Status (Intervention Groups Only)*

| Variable | Attended  0 Seminars | | Attended  ≥1 Seminar | |  | |  | |
| --- | --- | --- | --- | --- | --- | --- | --- | --- |
|  | ***M*** | ***SD*** | ***M*** | ***SD*** | ***t*** | ***p*** | | ***d*** |
| Child age (years) | 7.43 | 2.92 | 6.52 | 3.29 | 1.16 | .25 | | .29 |
|  | ***n*** | **%** | ***n*** | **%** | ***χ^2^*** | ***p*** | | ***V*** |
| Child Gender |  |  |  |  | 1.44 | .23 | | .14 |
| Male | 11 | 47.8 | 35 | 62.5 |  |  | |  |
| Female | 12 | 52.2 | 21 | 37.5 |  |  | |  |
| Child Ethnicity |  |  |  |  | 0.86 | .36 | | .10 |
| Hispanic/Latine | 18 | 78.3 | 38 | 67.9 |  |  | |  |
| Not Hispanic/Latine | 5 | 21.7 | 18 | 32.1 |  |  | |  |
| Child Race |  |  |  |  | 5.55 | .35 | | .27 |
| White | 13 | 59.1 | 38 | 69.1 |  |  | |  |
| Black | 6 | 27.3 | 8 | 14.5 |  |  | |  |
| Asian | 0 | 0.0 | 2 | 3.6 |  |  | |  |
| American Indian or Alaska Native | 0 | 0.0 | 2 | 3.6 |  |  | |  |
| More than one race | 2 | 9.1 | 1 | 1.8 |  |  | |  |
| Other | 1 | 4.5 | 4 | 7.3 |  |  | |  |

*Note.* Attended 0 Seminars (*n* = 23). Attended ≥1 Seminar (*n* = 56). *d* = Cohen’s d. V = Cramér’s V.

# **Table S9**

*Household Characteristics by Seminar Attendance Status (Intervention Groups Only)*

| Variable | Attended  0 Seminars | | Attended  ≥1 Seminar | |  |  |  |
| --- | --- | --- | --- | --- | --- | --- | --- |
|  | ***M*** | ***SD*** | ***M*** | ***SD*** | ***t*** | ***p*** | ***d*** |
| Number of children in the household | 1.91 | 0.95 | 2.07 | 1.01 | -0.65 | .52 | -.16 |
| Number of adults in the household | 1.74 | 0.45 | 1.89 | 0.41 | -1.47 | .15 | -.36 |
| Household income | $130,700 | $49,978 | $98,059 | $94,773 | 1.03 | .31 | .38 |
|  | ***n*** | **%** | ***n*** | **%** | ***χ^2^*** | ***p*** | ***V*** |
| Other Child Caregiver in the home |  |  |  |  | 3.93 | .05 | .22 |
| Yes | 12 | 52.2 | 42 | 75.0 |  |  |  |
| No | 11 | 47.8 | 14 | 25.0 |  |  |  |
| Local Address |  |  |  |  | 9.52 | .002 | .35 |
| Yes | 4 | 17.4 | 25 | 44.6 |  |  |  |
| No | 19 | 82.6 | 31 | 55.4 |  |  |  |
| Rural Address |  |  |  |  | .004 | .95 | .01 |
| Yes | 3 | 17.6 | 9 | 18.4 |  |  |  |
| No | 14 | 82.4 | 40 | 81.6 |  |  |  |

Attended 0 Seminars (*n* = 23). Attended ≥1 Seminar (*n* = 56). *d* = Cohen’s d. V = Cramér’s V. Local address incudes all parents who listed a mailing address in the city in which the research team was located. Rural address includes participants who provided a street address and zip code that was considered to be rural according to the Health Resources & Services Administration.

# **Table S10**

*Baseline Differences in Intervention Outcomes by Seminar Attendance Status (Intervention Groups Only)*

| Variable | Attended  0 Seminars | | Attended  ≥1 Seminar | |  | |  |
| --- | --- | --- | --- | --- | --- | --- | --- |
|  | ***M*** | ***SD*** | ***M*** | ***SD*** | ***t*** | ***p*** | ***d*** |
| Positive Parenting | 67.69 | 6.55 | 66.79 | 5.69 | 0.62 | .54 | .15 |
| Parental Warmth | 12.82 | 2.06 | 13.09 | 1.61 | -0.61 | .55 | -.15 |
| Parental Support | 13.17 | 1.70 | 12.84 | 1.55 | 0.85 | .40 | .21 |
| Positive Reinforcement | 17.61 | 2.15 | 17.02 | 2.14 | 1.12 | .27 | .28 |
| Proactive Parenting | 24.09 | 2.68 | 23.84 | 2.46 | 0.40 | .69 | .10 |
| Negative Parenting | 45.57 | 10.71 | 41.90 | 9.74 | 1.48 | .14 | .37 |
| Parental Hostility | 18.35 | 5.01 | 18.36 | 4.50 | -0.01 | .99 | .00 |
| Physical Control | 8.91 | 4.59 | 7.40 | 3.36 | 1.62 | .11 | .40 |
| Lax Control | 18.30 | 3.64 | 16.12 | 5.23 | 1.82 | .07 | .45 |
| Parental Psychological Control | 15.78 | 6.28 | 13.25 | 5.01 | 1.88 | .06 | .47 |
| Parental Knowledge (%) | 43.85 | 17.44 | 49.46 | 14.81 | -1.45 | .15 | -.36 |
| Parental Hope | 34.61 | 4.62 | 35.36 | 4.14 | -0.71 | .48 | -.18 |
| Readiness for Change | 26.70 | 4.13 | 27.18 | 3.24 | -0.56 | .58 | -.14 |
| Parental Self-Efficacy | 19.39 | 2.68 | 19.95 | 3.65 | -0.66 | .51 | -.16 |

*Note.* Attended 0 Seminars (*n* = 23). Attended ≥1 Seminar (*n* = 56). *d* = Cohen’s d. Parental knowledge is measured as the percentage of the items that parents got correct.

# **Table S11**

*Effects of Intervention on Positive Parenting Behaviors (Including Non-Attenders Assigned to Intervention Condition)*

|  | **Random Intercept** | | | | **Fixed Linear Time Slope** | | | |
| --- | --- | --- | --- | --- | --- | --- | --- | --- |
|  | *b* | *SE* | *p* | *d* | *b* | *SE* | *p* | *d* |
| **Positive Parenting** |  |  |  |  |  |  |  |  |
| Intercept | **67.17** | **0.99** | **< .001** | **10.93** | –– | –– | –– | –– |
| Time | –– | –– | –– | –– | –0.63 | 0.38 | .10 | –0.38 |
| 90-Minute Condition | –0.27 | 1.40 | .85 | –0.03 | **1.11** | **0.53** | **.04** | **0.47** |
| 60-Minute Condition | 0.10 | 1.43 | .94 | 0.01 | 0.86 | 0.52 | .10 | 0.37 |
| **Positive Reinforcement** |  |  |  |  |  |  |  |  |
| Intercept | **17.28** | **0.34** | **< .001** | **8.14** | –– | –– | –– | –– |
| Time | –– | –– | –– | –– | –0.17 | 0.13 | .20 | –0.30 |
| 90-Minute Condition | –0.23 | 0.48 | .63 | –0.08 | **0.53** | **0.19** | **.01** | **0.62** |
| 60-Minute Condition | 0.08 | 0.49 | .87 | 0.03 | 0.28 | 0.19 | .13 | 0.34 |
| **Parental Warmth** |  |  |  |  |  |  |  |  |
| Intercept | **12.89** | **0.27** | **< .001** | **7.95** | –– | –– | –– | –– |
| Time | –– | –– | –– | –– | –0.08 | 0.10 | .39 | –0.21 |
| 90-Minute Condition | 0.05 | 0.38 | .90 | 0.02 | 0.17 | 0.14 | .21 | 0.29 |
| 60-Minute Condition | 0.23 | 0.39 | .54 | 0.10 | 0.12 | 0.13 | .36 | 0.21 |
| **Parental Support** |  |  |  |  |  |  |  |  |
| Intercept | **13.00** | **0.25** | **< .001** | **8.38** | –– | –– | –– |  |
| Time | –– | –– | –– | –– | –0.03 | 0.10 | .78 | –0.07 |
| 90-Minute Condition | 0.10 | 0.35 | .77 | 0.05 | 0.10 | 0.14 | .45 | 0.17 |
| 60-Minute Condition | –0.21 | 0.36 | .56 | –0.09 | 0.01 | 0.13 | .96 | 0.01 |
| **Proactive Parenting** |  |  |  |  |  |  |  |  |
| Intercept | **24.01** | **0.45** | **< .001** | **8.28** | –– | –– | –– | –– |
| Time | –– | –– | –– | –– | –0.34 | 0.19 | .07 | –0.39 |
| 90-Minute Condition | –0.18 | 0.64 | .78 | –0.05 | 0.30 | 0.26 | .25 | 0.24 |
| 60-Minute Condition | 0.01 | 0.65 | .99 | 0.00 | 0.44 | 0.26 | .09 | 0.35 |

*Note.* Waitlist control group is the reference. Bold estimates represent statistically significant paths (*p* < .05). *d* = partial Cohen’s *d*.

# **Table S12**

*Effects of Intervention on Negative Parenting Behaviors (Including Non-Attenders Assigned to Intervention Condition)*

|  | **Random Intercept** | | | | **Fixed Linear Time Slope** | | | |
| --- | --- | --- | --- | --- | --- | --- | --- | --- |
|  | *b* | *SE* | *p* | *d* | *b* | *SE* | *p* | *d* |
| **Negative Parenting** |  |  |  |  |  |  |  |  |
| Intercept | **42.63** | **1.41** | **< .001** | **4.70** | –– | –– | –– |  |
| Time | –– | –– | –– | –– | –0.90 | 0.62 | .14 | –0.34 |
| 90-Minute Condition | –1.00 | 2.00 | .62 | –0.08 | –1.70 | 0.86 | .05 | –0.44 |
| 60-Minute Condition | 1.64 | 2.04 | .42 | 0.13 | –0.77 | 0.85 | .36 | –0.20 |
| **Parental Hostility** |  |  |  |  |  |  |  |  |
| Intercept | **18.13** | **0.69** | **< .001** | **4.03** | –– | –– | –– | –– |
| Time | –– | –– | –– | –– | –0.20 | 0.31 | .54 | –0.14 |
| 90-Minute Condition | –0.07 | 0.97 | .94 | -0.01 | –**0.95** | **0.44** | **.03** | –**0.47** |
| 60-Minute Condition | 0.48 | 0.99 | .63 | 0.07 | –0.29 | 0.43 | .51 | -–0.14 |
| **Physical Control** |  |  |  |  |  |  |  |  |
| Intercept | **7.20** | **0.53** | **< .001** | **2.39** | –– | –– | –– |  |
| Time | –– | –– | –– | –– | –0.21 | 0.14 | .13 | –0.38 |
| 90-Minute Condition | 0.54 | 0.76 | .48 | 0.13 | –0.15 | 0.20 | .44 | –0.19 |
| 60-Minute Condition | 0.80 | 0.77 | .31 | 0.18 | –0.03 | 0.20 | .89 | –0.03 |
| **Lax Control** |  |  |  |  |  |  |  |  |
| Intercept | **17.32** | **0.70** | **< .001** | **3.94** | –– | –– | –– | –– |
| Time | –– | –– | –– | –– | –0.42 | 0.28 | .13 | –0.35 |
| 90-Minute Condition | –1.47 | 0.99 | .14 | -0.24 | –0.56 | 0.39 | .15 | –0.32 |
| 60-Minute Condition | 0.41 | 1.01 | .69 | 0.07 | –0.43 | 0.38 | .27 | –0.25 |
| **Psychological Control** |  |  |  |  |  |  |  |  |
| Intercept | **0.20** | **-0.31** | **< .001** | **2.95** | –– | –– | –– | –– |
| Time | –– | –– | –– | –– | 0.00 | 0.27 | .99 | 0.00 |
| 90-Minute Condition | 0.71 | 1.03 | .49 | 0.11 | –0.49 | 0.38 | .20 | –0.31 |
| 60-Minute Condition | 1.29 | 1.05 | .22 | 0.20 | –0.72 | 0.38 | .06 | –0.45 |

*Note.* Waitlist control group is the reference. Bold estimates represent statistically significant paths (*p* < .05). *d* = partial Cohen’s *d*.

# **Table S13**

*Effects of Intervention on Parental Knowledge, Parental Hope, Readiness for Change, and Parental Self-Efficacy (Including Non-Attenders Assigned to Intervention Condition)*

|  | **Random Intercept** | | | | **Fixed Linear Time Slope** | | | |
| --- | --- | --- | --- | --- | --- | --- | --- | --- |
|  | *b* | *SE* | *p* | *d* | *b* | *SE* | *p* | *d* |
| **Parental Knowledge** |  |  |  |  |  |  |  |  |
| Intercept | **0.46** | **0.02** | **< .001** | **3.09** | –– | –– | –– | –– |
| Time | –– | –– | –– | –– | 0.01 | 0.01 | .15 | 0.33 |
| 90-Minute Condition | 0.02 | 0.03 | .47 | 0.12 | 0.01 | 0.01 | .40 | 0.19 |
| 60-Minute Condition | 0.02 | 0.04 | .54 | 0.10 | 0.02 | 0.01 | .05 | 0.44 |
| **Parental Hope** |  |  |  |  |  |  |  |  |
| Intercept | **35.78** | **0.58** | **< .001** | **10.12** | –– | –– | –– |  |
| Time | –– | –– | –– | –– | 0.13 | 0.22 | .57 | 0.14 |
| 90-Minute Condition | –0.09 | 0.82 | .91 | –0.02 | 0.41 | 0.31 | .19 | 0.32 |
| 60-Minute Condition | –1.20 | 0.83 | .15 | –0.24 | 0.55 | 0.31 | .08 | 0.42 |
| **Readiness for Change** |  |  |  |  |  |  |  |  |
| Intercept | **26.92** | **0.53** | **< .001** | **8.13** | –– | –– | –– | –– |
| Time | –– | –– | –– | –– | –0.14 | 0.21 | .51 | -0.16 |
| 90-Minute Condition | 0.44 | 0.75 | .56 | 0.09 | 0.16 | 0.29 | .59 | 0.13 |
| 60-Minute Condition | –0.20 | 0.77 | .79 | –0.04 | 0.39 | 0.29 | .18 | 0.31 |
| **Parental Self-Efficacy** |  |  |  |  |  |  |  |  |
| Intercept | **20.47** | **0.46** | **< .001** | **7.48** | –– | –– | –– | –– |
| Time | –– | –– | –– | –– | 0.29 | 0.17 | .09 | 0.43 |
| 90-Minute Condition | –0.52 | 0.64 | .42 | –0.14 | 0.30 | 0.24 | .21 | 0.31 |
| 60-Minute Condition | –0.74 | 0.66 | .26 | –0.19 | 0.26 | 0.24 | .28 | 0.27 |

*Note.* Waitlist control group is the reference. Bold estimates represent statistically significant paths (*p* < .05). *d* = partial Cohen’s *d*.
